# Functional Materials for Biosensing Applications

**DOI:** 10.3390/bios16080416

**Published:** 2026-08-03

**Authors:** Jin-Ha Choi

**Affiliations:** School of Chemical Engineering, Clean Energy Research Center, Jeonbuk National University, 567 Baekje-daero, Deokjin-gu, Jeonju-si 54896, Jeonbuk State, Republic of Korea; jhchoi@jbnu.ac.kr

A biosensor converts a biological recognition event into a measurable signal, but its analytical performance is determined as much by the material interface as by the recognition element itself. Surface chemistry controls immobilization and nonspecific adsorption; electronic and optical properties govern signal transduction and amplification; catalytic activity sets reaction efficiency; and mechanical behavior determines whether a device remains reliable in flexible, wearable, or portable formats [1,2].

Consequently, functional materials are no longer passive supports. Self-assembled polymers, metallic and carbon nanostructures, metal oxides, two-dimensional materials, catalytic nanozymes, and responsive coatings can be engineered to increase an effective surface area, localize electromagnetic fields, accelerate charge transfers, and couple molecular recognition to simple readouts. Their integration with microfluidic and miniaturized systems is moving biosensing toward decentralized testing, while simultaneously exposing persistent challenges in reproducibility, antifouling, long-term stability, scalable manufacture, and validation with real samples [3,4].

The Special Issue “Functional Materials for Biosensing Applications” was organized to capture this material-centered view of biosensor development. It brings together contributions spanning electrochemical, optical, colorimetric, and gas-sensing strategies, along with a broader discussion of engineered nanomaterials as biointerfaces for controlling stem cell behavior. A total of eight papers—one original research article and seven reviews—were published in the Special Issue.

Chung and Oh (contribution 1) review block copolymers as bottom-up materials for biosensing. They explain how molecular composition and self-assembly can control nanoscale orientation, shape, and periodicity, and then survey block-copolymer-based sensing platforms and the remaining limitations that must be addressed for broader analytical use.

Han et al. (contribution 2) report a flexible electrochemical biosensor that combines a lithography-assisted nanostructured polymer electrode with a mismatched-DNA/metal-ion-mediated recognition strategy. The platform selectively detects HPV-16 and HPV-18 nucleic acids without target amplification or chemical labeling, while retaining the conductive and nanostructural characteristics needed for operation under bending.

Kim et al. (contribution 3) examine nanomaterials for modulating stem cell differentiation and supporting regenerative applications. Their review covers metal-, carbon-, and peptide-based systems, extracellular-matrix-mimicking scaffolds, control of reactive oxygen species and autophagy, electrical stimulation, and nanomaterial-enabled delivery of biochemical or genetic cues.

Lee et al. (contribution 4) survey enzyme-stabilized gold nanoclusters, in which enzymatic specificity is combined with the optical and electrochemical properties of ultrasmall gold structures. They discuss preparation routes, sensing mechanisms, representative analytical applications, and the stability and performance issues that shape future development.

Kiejzik et al. (contribution 5) review exhaled aldehydes and ketones as noninvasive biomarkers of lung cancer and diabetes. They compare gas-sensor and electronic-nose technologies, including nanostructured receptor layers, molecularly imprinted materials, and biological recognition elements, and highlight the need for stable coatings, standardized sampling, and clinically reproducible classification.

Rajkumar et al. (contribution 6) assess electrochemical methods for determining resorcinol, an environmentally and biologically relevant phenolic compound. The review organizes progress in electrode modification with graphene derivatives, metal–organic frameworks, MXenes, metal oxides, conductive polymers, and hybrid composites, together with the voltammetric and amperometric techniques used for quantification.

Kim et al. (contribution 7) focus on plasmonic biosensors for cancer-associated microRNA detection. They review recent surface-plasmon-resonance, localized-surface-plasmon-resonance, and plasmon-enhanced approaches, emphasizing engineered nanomaterials and amplification schemes that may translate low-abundance microRNA signatures into practical liquid-biopsy readouts.

Lee et al. (contribution 8) provide a broad review of colorimetric biosensors enabled by functional nanomaterials. Their discussion links material-dependent color-generation mechanisms with signal amplification, nanozyme catalysis, portable imaging, and artificial-intelligence-assisted analysis, showing how visually readable assays can evolve toward quantitative point-of-care and self-testing systems.

Taken together, these contributions show that the most useful sensing materials connect a well-defined molecular function to a manufacturable device architecture. Future progress will require reference standards, batch-to-batch consistency, robust antifouling, clinically or environmentally representative samples, scalable fabrication, multiplexed measurement, and portable readers with transparent data-analysis workflows. We hope that this collection will support those efforts and stimulate further collaboration across materials science, analytical chemistry, engineering, and biomedicine.
